# Assessment of the Impact of a Daily Rehabilitation Program on Anxiety and Depression Symptoms and the Quality of Life of People with Mental Disorders during the COVID-19 Pandemic

**DOI:** 10.3390/ijerph18041434

**Published:** 2021-02-03

**Authors:** Joanna Smolarczyk-Kosowska, Anna Szczegielniak, Mateusz Legutko, Adam Zaczek, Łukasz Kunert, Magdalena Piegza, Robert Pudlo

**Affiliations:** 1 Department of Psychiatry, Faculty of Medical Sciences in Zabrze, Medical University of Silesia, 42-612 Katowice, Poland; psychiatriatarnowskiegory@sum.edu.pl (M.L.); adam.zaczek@med.sum.edu.pl (A.Z.); lkunert@sum.edu.pl (Ł.K.); mpiegza@sum.edu.pl (M.P.); rpudlo@sum.edu.pl (R.P.); 2 Department of Psychiatric Rehabilitation, Department of Psychiatry and Psychotherapy, Faculty of Medical Sciences in Katowice, Medical University of Silesia, 40-635 Katowice, Poland; anna.szczegielniak@med.sum.edu.pl

**Keywords:** COVID-19, community psychiatry, psychiatric rehabilitation, anxiety, depression, quality of life

## Abstract

Community psychiatry is a modern and effective form of care for patients with mental disorders. The aim of the study was to assess the impact of a rehabilitation program at the Mental Health Support Centre in Tarnowskie Góry (Poland) on reducing severity of anxiety and depression symptoms, as well as improving overall quality of life during the COVID-19 pandemic. The study involved 35 patients, examined with an authors’ questionnaire on sociodemographic data, the Hospital Scale of Anxiety and Depression (HADS) and the Short Form Health Survey (SF-36). Data was obtained during the first national lockdown and compared to data gathered before the pandemic on the same study group. Imposed restrictions, negative emotional state during lockdown, subjectively assessed higher health risk and a low level of knowledge about the COVID-19 pandemic did not significantly correlate with a severity of depression and anxiety, as well as general quality of life. However, the comparison of the results obtained in HADS and SF-36 scales show a significant improvement in both categories. Rehabilitation activities, including physical training, cognitive exercise and social therapy, reduce the severity of the symptoms and have a positive effect on the overall quality of life in patients suffering from schizophrenia and affective disorders. Therefore, holistic mental health support services may positively affect building an individual resilience. The severity of anxiety symptoms during the COVID-19 pandemic shows a negative correlation with the patient’s age.

## 1. Introduction

Community psychiatry, being a modern and effective form of care for patients with mental disorders, is widely implemented across the world and taking various forms depending on the needs of a given community. Research results confirming the positive effects of introducing integrated hospital and community psychiatric services were published as early as in the 1980s [1]. It has been shown that when the reduction of hospital beds is done responsibly the total costs of community care services are similar to those of hospital services for long-term patients, while the quality of life and satisfaction of those receiving community care are higher than those of those treated in hospital [2]. The World Psychiatric Association (WPA) defines community psychiatry through different practices and assumptions such as addressing population needs in ways that are accessible and acceptable; building on the goals and strengths of people who experience mental illnesses; promoting a wide network of supports, services and resources of adequate capacity and emphasizing services that are both evidence-based and recovery-oriented [3]. Large variations in the implementation of community mental health services can be currently observed across the world [4]. Social support is an important yet missing element in terms of holistic approach among those receiving formal psychiatric services [5]. In the early years of the 21st century, the World Health Organization recommended patients to be transferred from psychiatric hospitals and other long-stay facilities to institutions providing care within the framework of community psychiatry [6]. According to this recommendation, increasingly, facilities are being created with services based on the model of community treatment.

In 2019, the Mental Health Support Centre (Centrum Wsparcia Zdrowia Psychicznego, CWZP) in Tarnowskie Góry (Poland) was established. As a part of this project, variety of pro-health activities, aimed at preserving, restoring and improving the mental health of people suffering from mental disorders, were introduced. CWZP patients participate in a three-month original rehabilitation program created in response to the needs of the participants. Its elements are: Walks, gymnastics, individual exercises with a physiotherapist, art therapy, music therapy, culinary training, relaxation, yoga, cognitive training including computer-based programs (Neuroforma, CogniPlus, RehaCom), psychological workshops, group classes, individual meetings with a psychologist and consultations with a psychiatrist. The Patient Activity Card is included in Supplementary Materials. The intensity of physical exercise and cognitive training depends on the initial condition and predisposition of the patient, which are individually assessed by the physiotherapist and the physician at the beginning of the program. Patients are qualified for the physiotherapy program on the basis of selected elements of the Fullerton test, which is considered particularly useful in the multivariate assessment of physical fitness in elderly people (six-min walk test—6MWT with the assessment of the degree of fatigue in the modified Borg scale, Back Scratch test, Up and go test, Chair Sit-and-Reach test). During the qualification, patients also undergo the Romberg test to assess possible balance and gait disturbance. The patients’ physical fitness is assessed regularly also on the 6th and 12th week of stay at the CWZP. The goals and rehabilitation plan are defined individually on the basis of the results achieved in the tests and the expectations of patients. The treatment program includes group exercises to improve physical fitness, breathing exercises and relaxation with elements of body awareness exercises. In March 2020, the activities of CWZP were temporarily suspended due to the COVID-19 outbreak. 

There are reports in the literature confirming that both the pandemic itself and the government-driven measures taken to combat it may negatively affect the mental health of the patients [7]. Subjecting people with mental disorders to compulsory restrictions may result in reinforcement of anxiety and depressive symptoms, exposure of obsessive behaviour, and, in the longer term, development of a post-traumatic stress disorder [8]. The impact of a pandemic on mental health can depend on many variables, such as gender, age, place of residence, the presence of a mental illness diagnosed before or any other chronic diseases, and the incidence of COVID-19 among family or friends. The results of a study conducted in Turkey in April 2020 indicate that women, inhabitants of urban areas, people with a previously diagnosed mental illness and those suffering from other chronic diseases are the most vulnerable to the negative effects of the COVID-19 pandemic [7].

These reports inspired the authors of this study to attempt an assessment of the impact of the risk connected to the SARS-CoV-2 pandemic on the mental health of the CWZP patients and the effectiveness of implemented rehabilitation program, both in reducing the intensity of negative emotions and improving the quality of life.

The aim of the study was to assess the impact of the holistic rehabilitation program at the Mental Health Support Centre in Tarnowskie Góry on reducing severity of anxiety and depression symptoms and improving the overall quality of life during the difficult psychosocial situation in due to the COVID-19 pandemic.

## 2. Materials and Methods

The survey was conducted among patients of the Mental Health Support Centre in Tarnowskie Góry, who started the rehabilitation program in the period between December 2019 and February 2020. Patients were individually selected for the project. The time of recruitment to the program and its start depended on the psychophysical condition of patients and the availability of places in CWZP. The study involved 35 patients diagnosed with schizophrenia, affective disorders, anxiety disorders or organic mental disorders meeting the other criteria for inclusion in the program: 18 years of age, psychophysical condition enabling safe use of the activities offered and ability to give an informed consent to participate in the project. The exclusion criteria were: addiction to alcohol or other psychoactive substances with the inability to maintain a 3-month abstinence or breaking abstinence during the project, withdrawing consent to participate in the project or deterioration of health preventing further use of rehabilitation activities.

Patients were examined through a form consisting of questions on sociodemographic data and two standardized measurement scales: the Hospital Scale of Anxiety and Depression (HADS), used to measure the severity of depressive and anxiety symptoms, and the Short Form Health Survey (SF-36) assessing the overall quality of life.

**Figure 1:**
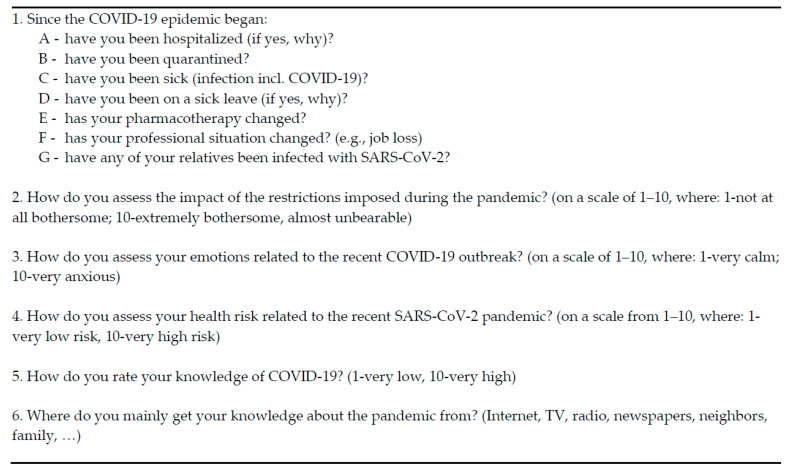
Questions regarding the situation of patients during the COVID-19 pandemic.

From the study group, 6 patients completed the entire 3-month rehabilitation program. The remaining 29 patients faced discontinuity of their participation in the program due to the suspension of the CWZP in the exceptional epidemiological situation. According to the project assumptions, the first mental state assessment took place when the therapeutic process was started. After the Centre’s activities were suspended, all patients were subjected to another survey. The project participants re-completed the HADS and SF-36 questionnaires and, additionally, the original form containing questions about the situation of patients during the COVID-19 pandemic (Figure 1) between 6 and 8 weeks after the end or interruption of the therapeutic cycle. Therefore, the mean duration of the research period of observation was 14.5 weeks.

The Hospital Scale of Depression and Anxiety (HADS) was created for the study of non-psychiatric patients aged 16–65 years. This scale consists of 14 questions, 7 of which are related to anxiety (HADS-A subscale) and 7 to depression (HADS-D subscale). Each question is rated on a scale of 0 to 3 points. The scoring range is the same for the HADS-A and HADS-D subscales. A higher score value corresponds to a greater severity of symptoms [9]. The HADS scale allows the assessment of anxiety and depression in both hospitalized patients and outpatient facilities’ users [10]. It is a scale appropriate for the initial diagnosis of depression and anxiety disorders and for the assessment of the severity or withdrawal of their symptoms [11]. The cut-off value is 7 points.

The SF-36 is a questionnaire intended for the subjective assessment of the quality of life. It consists of 36 statements that take into account: Physical functioning, limitations resulting from the state of physical health, pain, general sense of health, vitality, social functioning, emotional functioning and a sense of mental health. The quality of life index is the sum of the assessment points for all 8 domains and allows for a comprehensive assessment of health. The maximum possible points to gain is 171. According to the Polish version of the SF-36 questionnaire, the higher the score, the lower quality of life, and the lower the score, the higher quality of life [12,13].

The collected data was processed via the Statistica 13.3 program, licensed by the Medical University of Silesia in Katowice. The Shapiro-Wilk test was used to assess the normality of distributions. To compare quantitative variables, the Mann Whitney U test for a dichotomous grouping variable and ANOVA Kruskal-Wallis for a variable with a group number greater than 2 were used. The relationships between quantitative variables were assessed using the Spearman’s rank correlation coefficient. To compare the results obtained by patients in the HADS and SF-36 scales in the first and second measurements, the Student’s *t*-test for dependent samples in regards to variables with a normal distribution and the Wilcoxon test for variables with a deviation from the normal were used. We assumed *p* < 0.05 as statistically significant.

The study received a positive opinion of the Bioethics Committee of the Medical University of Silesia in Katowice.

## 3. Results

The study involved 35 patients of the Mental Health Support Center in Tarnowskie Góry, suffering from schizophrenia, affective disorders, anxiety disorders or organic mental disorders. There were 20 women and 15 men among the respondents. Somatic diseases were found in 27 subjects (77%), the most common of which were arterial hypertension (31.4%) and diabetes (28.6%). The sociodemographic data is presented in the table (Table 1). The mean duration of participation in the program was 33 days (normal distribution; mean = 33.0; sd = 15.93).

The analysis of the results obtained by patients in the HADS and SF-36 questionnaires showed correlation with age. In the HADS-A subscale (Spearman’s rank-order—R = −0.52; *p* < 0.05) and overall HADS score (R = −0.41; *p* < 0.05) greater severity of symptoms was noted in younger subjects. However, a similar relationship was not observed in the study with the use of SF-36, where age had no significant influence on the results obtained in the quality of life scale. On the other hand, a comparative analysis in terms of the place of residence showed significant differences in the assessment of the quality of life. People living in rural areas assessed their quality of life better than city dwellers (Kruskal-Wallis test—H = 10.19; *p* < 0.05).

In our study group antidepressants were taken by 19 patients; antipsychotics by 9 (low dose 4, medium dose 1, high dose 4); anxiolytics by 7; normotymics by 3; other drugs by 14 people. Psychotropic treatment of patients did not change during the research period.

None of the patients in the study were quarantined or hospitalized during the COVID-19 pandemic. No SARS-CoV-2 infection was identified in any of the patients or their family members. The imposed restrictions, emotions related to the pandemic, subjectively assessed significance of health risk and level of knowledge about the SARS-CoV-2 pandemic did not significantly affect the severity of depression and anxiety symptoms, as well as overall quality of life (Kruskal-Wallis test, *p* > 0.05, Table 2).

**Figure 2:**
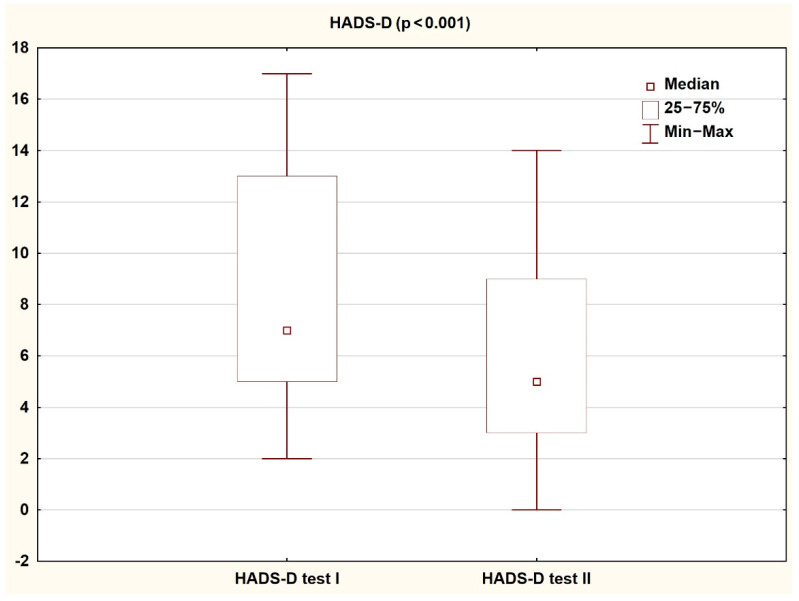
Patient results in the HADS-D subscale during the first and second study (Wilcoxon test).

**Figure 3:**
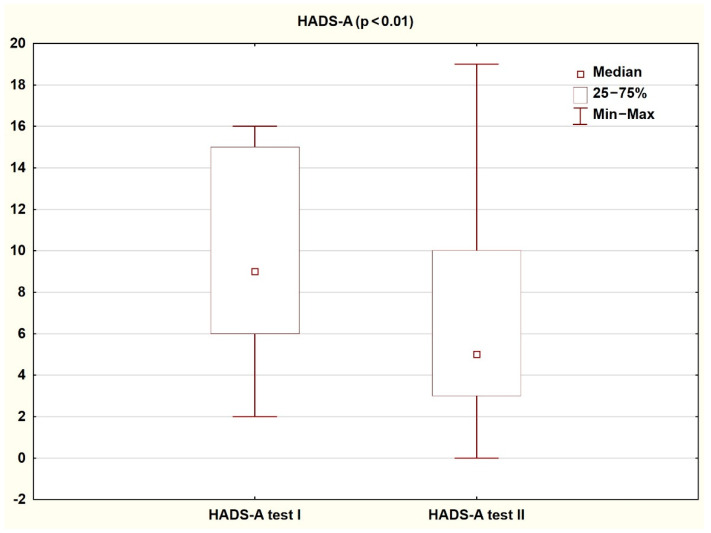
Patient results in the HADS-A subscale during the first and second study (Wilcoxon test).

**Figure 4:**
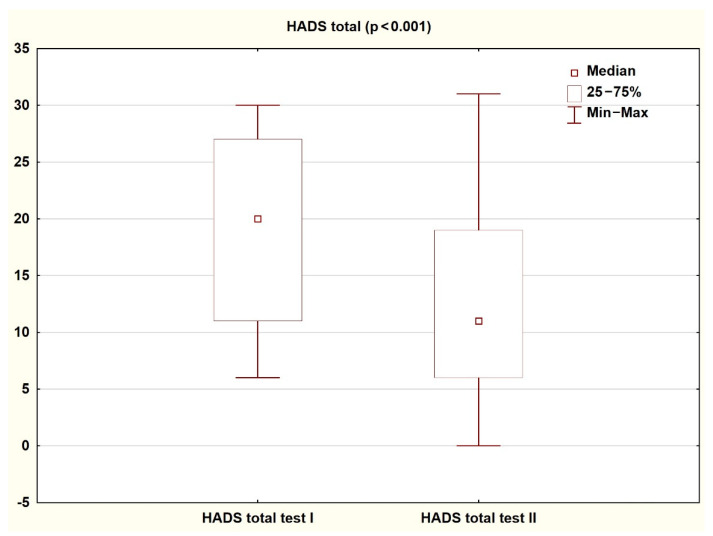
Patient results in the HADSscale during the first and second study (Wilcoxon test).

**Figure 5:**
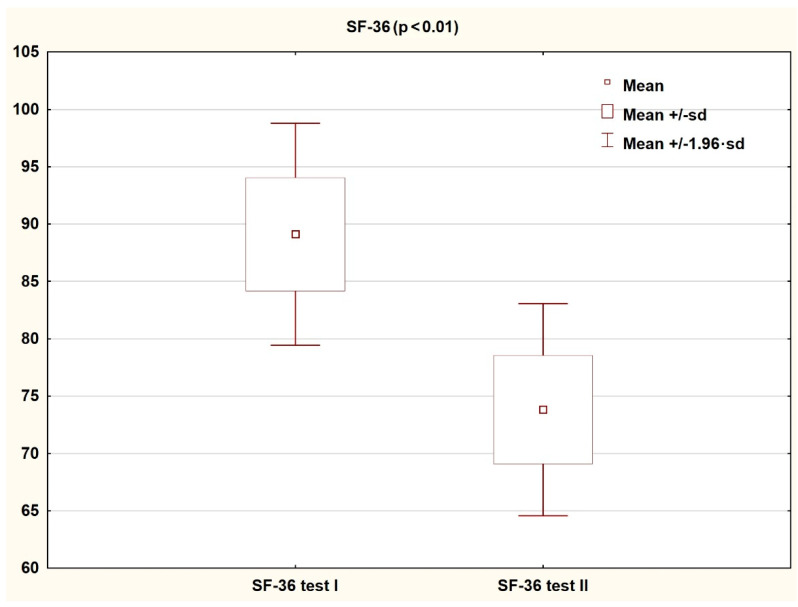
Patient results in the SF-36 scale during the first and second study (Student’s *t*-test for dependent variables).

Comparison of the results obtained by patients on the HADS and SF-36 scales in the first and second assessment shows a significant improvement in the quality of life and reduction in the severity of both depressive and anxiety symptoms (*p* < 0.05). The results are presented in the tables and figures (Table 3 and Table 4; Figure 2, Figure 3, Figure 4 and Figure 5).

The analysis of the results after taking into account the initial diagnosis showed a lower intensity of depressive and anxiety symptoms in all four groups of patients during the second assessment. A noticeable difference is observed in all subgroups, but not everywhere it reaches the level of statistical significance, which is most likely related to a small number of patients within them. The results of the quality of life assessment are similar, as it improved in all the studied subgroups, however, it achieved statistical significance only in the group of patients with affective disorders. The detailed analysis is presented in Table 5.

## 4. Discussion

The results of our study confirmed the hypothesis that active participation in a rehabilitation program at the Mental Health Support Centre reduces the severity of depression and anxiety symptoms, and has a positive effect on the overall quality of life of patients suffering from schizophrenia, affective disorders, anxiety disorders or organic mental disorders. The improvement is noticeable despite the fact that patients left the rehabilitation program before its completion (12 weeks) and were exposed to variety of burdens caused by the state of pandemic. However, we cannot exclude that their mental condition will worsen in the long term. According to the dynamics of the psychophysiological response to stress, mental mobilization during an epidemiological threat may cause the distant effects of trauma to appear in a few months or even years [14].

The severity of anxiety symptoms during the COVID-19 pandemic has a negative correlation with age. In our opinion, this may be caused by the greater activity of young people in social media. Unreliable information of a sensational nature transmitted through them may increase the level of fear and anxiety [15]. In a study of the German population, cyberchondria measured with the abbreviated version of the Cyberchondria Scale (CSS-15) positively correlates with the severity of anxiety during the COVID-19 pandemic [16]. However, this study did not show direct dependency between age and the level of cyberchondria, which prompts the search for other reasons that cause the relationship between young age and the severity of anxiety during a pandemic. It is possible that the reason is the greater fear among young people about the health of family members and about the economic effects of the pandemic, as suggested by both the results of the above-mentioned German study and the study conducted in Iran, where the highest level of anxiety was recorded among people aged 21–40 [16,17].

However, no significant differences in the severity of anxiety in correlation with gender were observed, which can be explained by the fact that although women are more prone to developing anxiety disorders, they also engage in more effective preventive behaviours during a pandemic [18], which may consequently increase the sense of self-efficacy and help reduce anxiety.

The most serious limitation is the small size of the study group, however, it should be noted that the project was temporarily discontinued due to the pandemic, which made it impossible to expand it. Another serious limitation of our work is a lack of a control group. Therefore, we cannot clearly state whether it was the participation in the rehabilitation program that improved the mental health of the respondents. It cannot be ruled out that the pandemic itself could be a factor that could have contributed to it. There are suggestions in the literature that for some patients the introduced restrictions are not burdensome at all, and may even turn out to be beneficial, e.g., in the case of people for whom the obligation to maintain social distance reduces the stress associated with the need to go out. from home and / or interacting with other people [19]. However, most of the studies conducted indicate a deterioration of the mental state due to the pandemic restrictions [20,21]. In the case of our subjects, the positive impact of the restrictions is also unlikely, as only 4 out of 35 patients, when asked about the restrictions related to the pandemic, replied that they were not burdensome for them at all. In addition, different main diagnoses of patients, thus, as a consequence, different etiology of the reported symptoms, could have influenced the obtained results.

## 5. Conclusions

1. Rehabilitation activities, including physical training, cognitive exercise and social therapy, can reduce the severity of depression and anxiety symptoms and have a positive effect on the overall quality of life in patients suffering from mental disorders.
2. The imposed restrictions, emotions related to the pandemic, subjectively assessed significance of health risk and level of knowledge about the SARS-CoV-2 pandemic did not significantly affect the severity of depression and anxiety symptoms, as well as overall quality of life.
3. The severity of anxiety symptoms during the COVID-19 pandemic shows a negative correlation with the patient’s age.

## Figures and Tables

**Table 1:** Study group characteristics.

**Age**				
**N**	**Median**	**Min.**	**Max.**	
35	66	23	85	
**Sex**				
	Women	Men		
	20 (57.14%)	15 (42.86%)		
**Diagnosis**				
	Schizophrenia	Affective disorders	Anxiety disorders	Organic mental disorders
	3 (8.57%)	6 (17.14%)	20 (57.14%)	6 (17.14%)
**Marital Status**				
	Single	Married	Divorced/ Separated	Widow/ Widower
	7 (20%)	7 (20%)	4 (11.43%)	17 (48.57%)
**Place of Residence**				
	Rural area	Urban area (up to 100,000 inhabitants)	Urban area (between 100,000 and 300,000 inhabitants)	Urban area (above300,000 inhabitants)
	6 (17.14%)	26 (74.29%)	2 (5.71%)	1 (2.86%)
**Housing Situation**				
	Living alone	Living with family/ relatives		
	20 (57.14%)	15 (42.86%)		
**Professional Activity**				
	Pension	Disablement/ Sickness pension	Other	
	22 (62.86%)	10 (28.57%)	3 (8.57%)	
**Smoking Cigarettes**				
	Yes	No		
	4 (11.43%)	31 (88.57%)		
**Comorbidities**				
	Hypertension	Diabetes	Diseases of the thyroid gland	Gout
	11 (31.43%)	10 (28.26%)	8 (22.9%)	3 (8.5%)

**Table 2:** Factors describing the situation of patients during the pandemic—statistical analysis—Kruskal-Wallis test.

	Restrictions	Emotions	Health Risk	Level of Knowledge
**HADS-total**	H = 7.21; *p* > 0.05	H = 9.69; *p* > 0.05	H = 14.76; *p* > 0.05	H = 3.14; *p* > 0.05
**SF-36**	H = 7.47; *p* > 0.05	H = 7.93; *p* > 0.05	H = 13.84; *p* > 0.05	H = 5.86; *p* > 0.05

**Table 3:** Results achieved by patients on the HADS scale during the first and second tests (Wilcoxon test).

	Study I			Study II				
	Median	Q1	Q3	Median	Q1	Q3	Z	P
**HADS-D**	7.00	5.00	13.00	5.00	3.00	9.00	3.57	<0.001
**HADS-A**	9.00	6.00	15.00	5.00	3.00	10.00	2.73	<0.01
**HADS Total**	20.00	11.00	27.00	11.00	6.00	19.00	3.53	<0.001

**Table 4:** Results achieved by patients on the SF-36 scale during the first and second tests (Student’s *t*-test).

	Mean	SD	N	T	Df	*p*	−95% Confidence-Interval	+95% Confidence- Interval
**SF-36 Test I**	89.11	29.24						
**SF-36 Test II**	73.83	27.95	35	3.05	34	0.004	5.095306	25.47612

**Table 5:** Results achieved by patients on the HADS and SF-36 scale during the first and second tests according to a diagnosis (Wilcoxon test).

**Schizophrenia**								
	**Study I**			**Study II**				
	**Median**	**Q1**	**Q3**	**Median**	**Q1**	**Q3**	**Z**	***P***
**HADS-D**	13.00	8.00	16.00	11.00	10.00	13.00	1.07	>0.05
**HADS-A**	14.00	9.00	16.00	8.00	8.00	18.00	0.53	>0.05
**HADS Total**	25.00	24.00	27.00	21.00	18.00	28.00	1.07	>0.05
**SF-36**	132.00	82.00	133.00	80.00	74.00	86.00	1.07	>0.05
**Affective Disorders**								
	**Study I**			**Study II**				
	**Median**	**Q1**	**Q3**	**Median**	**Q1**	**Q3**	**Z**	***P***
**HADS-D**	12.50	7.00	14.00	8.00	1.00	11.00	1.57	>0.05
**HADS-A**	14.00	11.00	16.00	10.00	3.00	18.00	0.94	>0.05
**HADS Total**	25.00	20.00	29.00	22.50	4.00	26.00	1.47	>0.05
**SF-36**	100.50	95.00	117.00	90.50	61.00	104.00	2.20	<0.05
**Anxiety Disorders**								
	**Study I**			**Study II**				
	**Median**	**Q1**	**Q3**	**Median**	**Q1**	**Q3**	**Z**	***P***
**HADS-D**	6.50	4.50	10.00	4.50	2.50	6.50	2.37	<0.05
**HADS-A**	7.50	5.00	13.00	5.00	3.00	9.00	1.92	0.055
**HADS Total**	13.00	10.50	23.50	11.50	5.50	13.00	2.39	<0.05
**SF-36**	82.50	57.50	97.50	80.00	45.50	93.00	1.57	>0.05
**Organic Mental Disorders**								
	**Study I**			**Study II**				
	**Median**	**Q1**	**Q3**	**Median**	**Q1**	**Q3**	**Z**	***P***
**HADS-D**	9.00	5.00	13.00	3.50	3.00	5.00	2.20	<0.05
**HADS-A**	8.00	4.00	14.00	4.50	3.00	5.00	1.75	0.07
**HADS Total**	20.00	9.00	24.00	9.00	6.00	10.00	2.20	<0.05
**SF-36**	77.50	60.00	107.00	67.00	54.00	82.00	1.68	0.09

## Data Availability

The data presented in this study are available on request from the corresponding author. The data are not publicly available due to the protection of personal data.

## Supplementary Materials

The following are available online at https://www.mdpi.com/1660-4601/18/4/1434/s1, PATIENT ACTIVITY CARD.
