# Supplementary material for: Assessment of the Impact of a Daily Rehabilitation Program on Anxiety and Depression Symptoms and the Quality of Life of People with Mental Disorders during the COVID-19 Pandemic
Source: Int J Environ Res Public Health. 2021 Feb 3;18(4):1434. doi: 10.3390/ijerph18041434 (PMC7913642; doi:10.3390/ijerph18041434)
Supplement: Supplementary file 1 [file ijerph-18-01434-s001.pdf]

PATIENT ACTIVITY CARD

Name and surname: ID number:

Week of stay: Dates:

Activity	Monday	Tuesday	Wednesday	Thursday	Friday
Psychiatric consultation					
Psychoeducation					
Education of families or guardians					
Therapeutic community					
Psychological examination					
Individual psychological therapy					
Group therapy with psychologist					
Soft skills training & activization exercises					
Relaxation					
Individual psychotherapy					
Morning gymnastics (group)					
Respiratory and general fitness training (group)					
Balance training (group)					
Interval training (individual)					
Choreo therapy					
Art therapy					
Horticulo therapy					

PATIENT ACTIVITY CARD

Name and surname: ID number:

Ludo therapy					
Music therapy					
Bibliotherapy					
Social skills training					
Individual consultation with a social worker					
Computer-based cognitive training					
Massage chair					
Culinary training					
Others:					

Patient`s signature:
